# Detecting Transient Trapping from a Single Trajectory: A Structural Approach

**DOI:** 10.3390/e23081044

**Published:** 2021-08-13

**Authors:** Yann Lanoiselée, Jak Grimes, Zsombor Koszegi, Davide Calebiro

**Affiliations:** 1 Institute of Metabolism and Systems Research, University of Birmingham, Birmingham B15 2TT, UK; JXG937@student.bham.ac.uk (J.G.); Z.Koszegi@bham.ac.uk (Z.K.); d.calebiro@bham.ac.uk (D.C.); 2 Centre of Membrane Proteins and Receptors (COMPARE), Universities of Nottingham and Birmingham, Birmingham B15 2TT, UK

**Keywords:** single particle trajectory, stochastic processes, trapping, confinement

## Abstract

In this article, we introduce a new method to detect transient trapping events within a single particle trajectory, thus allowing the explicit accounting of changes in the particle’s dynamics over time. Our method is based on new measures of a smoothed recurrence matrix. The newly introduced set of measures takes into account both the spatial and temporal structure of the trajectory. Therefore, it is adapted to study short-lived trapping domains that are not visited by multiple trajectories. Contrary to most existing methods, it does not rely on using a window, sliding along the trajectory, but rather investigates the trajectory as a whole. This method provides useful information to study intracellular and plasma membrane compartmentalisation. Additionally, this method is applied to single particle trajectory data of $\beta_{2}$-adrenergic receptors, revealing that receptor stimulation results in increased trapping of receptors in defined domains, without changing the diffusion of free receptors.

## 1. Introduction

Single particle methods, which track fluorescent molecules over time, allow for the quantification of biological events with unprecedented spatial and temporal resolution. In cell biology, the complex organisation of the plasma membrane significantly impacts the lateral diffusion of membrane proteins, leading to non-stationary motion patterns. A proper interpretation of these complex trajectories requires that we take into account the changes in a molecule’s underlying motion mechanism. For example, transient trapping of G-protein-coupled receptors and G-proteins is closely related to a restricted collision-coupling model [1,2]. In this model, the association rates of molecules on the plasma membrane are enhanced by the presence of confining nano-domains, where receptors and G-proteins are more likely to encounter one another. However, using analysis tools that assume the same molecular motion over time leads to incorrect interpretations of the underlying biology. An intermittent process alternating between free Brownian motion and trapping (as observed in [3]) can wrongly be interpreted as a case of anomalous diffusion with an anomalous exponent α<1.

In the present article, we introduce a method to detect transient trapping events within a single trajectory. An advantage of analysing transient trapping events is the possibility of quantifying the binding kinetics of a molecule through different cellular nano-domains. Additionally, this approach does not require multiple visits of independent molecules to the same nano-domain to assess trapping and does not assume trapping nano-domains to be long-lived. Our strategy is to isolate different trapped portions of trajectories by considering the spatial self-localization of consecutive points within a single trajectory. We introduce local measures computed for each trajectory point, n∈[1,N], containing information on neighbouring trajectory coordinates as a way to elucidate the structure of the trajectory. For each trajectory position, the number of neighbours considered for the local measure is determined by the number of consecutive trajectory coordinates within the range of the test lengthscale.

The detection of trapping is challenging and has been the subject of investigation by several authors. A possible strategy, based on an ensemble of trajectories, consists of evaluating trapping domains from the evaluation of local confining force [4,5,6,7,8]. On the side of single trajectory analysis, techniques were based on the maximum square displacement [9,10,11], although they are generally too sensitive to noise and local fluctuations of trajectory dynamics. Following this, a number of methods were developed, including: image analysis techniques [12], a model specific maximum likelihood estimator [13], random forest models [14], back propagation neural network approaches [15], moment-scaling spectrum analysis [16], and standardized maximum distance [17]. Another approach proposes to detect confinement size based on first-passage times [18]. Closer to our approach, Sikora et al. [19,20] have developed a method for transient confinement identification based on recurrence statistics, and Verdier et al. [21] used a graphical representation of trajectories to identify the diffusion mode of a whole trajectory. Most of the above-mentioned techniques [9,10,11,14,15,16,19,20] rely on time window approaches. Alternatively, our method is based on a recurrence matrix and investigates a trajectory as a whole, whilst still determining sub-trajectory dynamics.

Recurrence matrices are used in various areas of science. They can be used to reconstruct protein structure [22] and are even used to detect structural changes in reaction-diffusion systems [23]. In general, they are used for quantifying non-linear time-series derived from dynamical systems, such as detecting protein conformation changes in molecular dynamics [24] or for quantifying physiological measurements [25]. In the context of dynamical systems, it has been shown that one can reconstruct the chaotic attractor associated with a time-series [26]. Additionally, the influence of observational noise on recurrence plots has been previously investigated [27], in addition to recurrence plots being used for testing time-series stationarity [28]. Although the concept of a recurrence matrix is not new, we construct it in a modified way that greatly limits the effect of outliers and localisation error. Our central hypothesis is that a trapping event within a trajectory is translated into a recurrence matrix as a square block structure along the diagonal of the recurrence matrix. We introduce 3 new local measures that are particularly relevant in detecting block structures along the diagonal, which are characteristic signatures of molecular trapping. From both our construction and these newly introduced measures, we derive a quantity that is invariant when the molecule is trapped and close to zero everywhere else.

In Section 3, we performed extensive simulations and tests to assess the reliability of our method and its robustness to noise for both 2D and 3D trajectories, comparing our method to the ‘Divide and Conquer Moment Scaling Spectrum’ (DC-MSS) [16]. Finally, in Section 4, we apply our method to single particle tracking data to trajectories of a prototypical G-protein-coupled receptor ($\beta_{2}$ adrenergic receptor), analysing the effects of different pharmacological treatments on receptor trapping.

## 2. Methods

**Figure 1:**
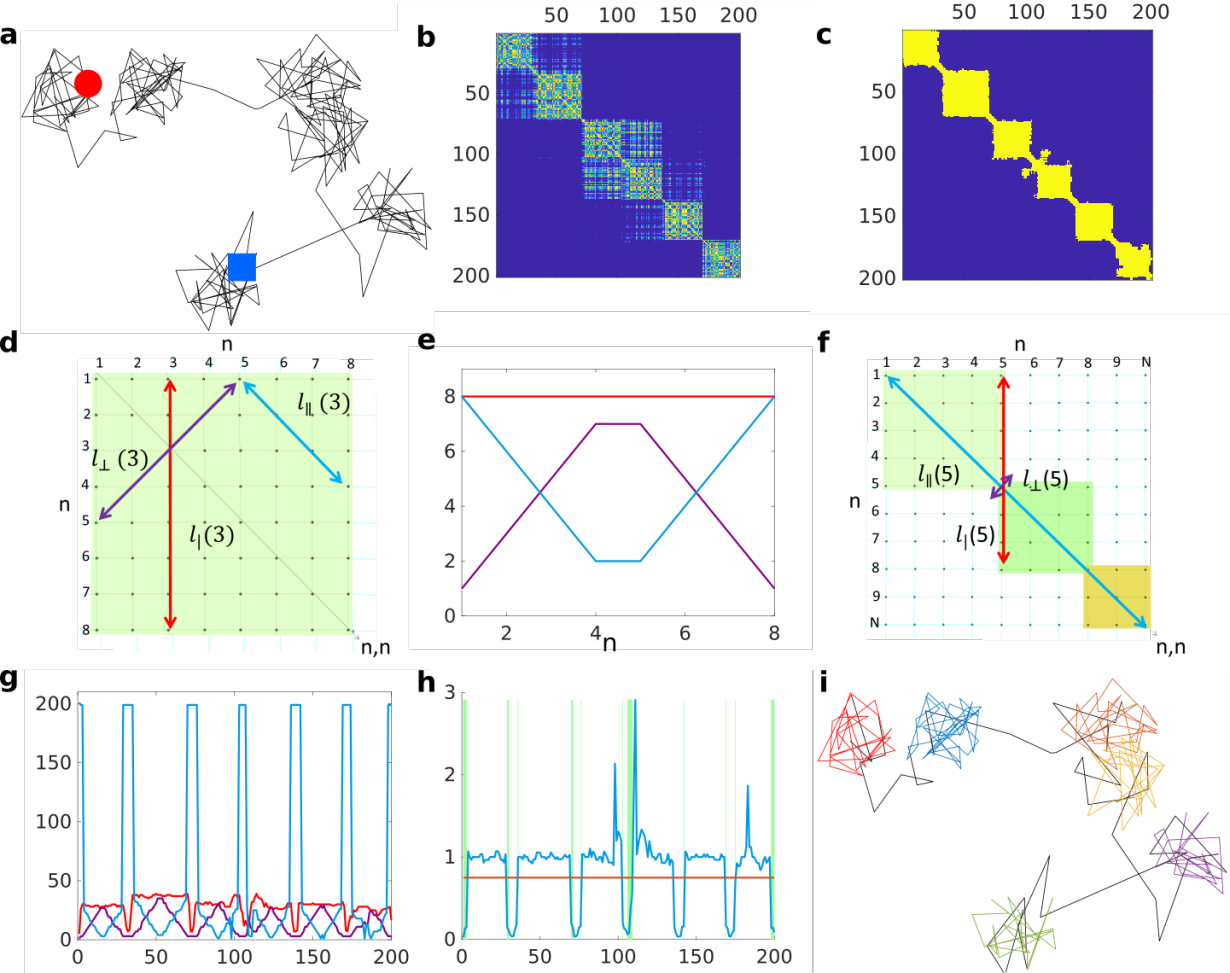
(**a**). Simulated 2D trajectory alternating between free Brownian motion and reflected diffusion in a disk of radius R=1. Diffusion coefficient is D=1/2 in both cases and duration of states in both cases is a Poisson distributed duration with mean $T_{f}=5$ and $T_{f}=30$ for free and reflected motions, respectively. Red circle denotes beginning and blue square the end of the trajectory. (**b**). Matrix *M* computed from trajectory in (**a**) with a test lengthscale λ=1. (**c**). Binary matrix *B* after thresholding *M* in (**b**), filling the lacunarities and adding diagonal lines. (**d**). Illustration of the the block time $t_{|}$ (red), the neighbouring time $t_{⊥}\left(n\right)$ (purple), and the persistence time $t_{\|}\left(n\right)$ (cyan) computed at the step n=3 of an ideal *B* matrix illustrating a fully trapped trajectory of 8 steps. (**e**). Illustration of the inequality along the diagonal $B_{nn}$ for a perfect block $t_{|}=t_{\|}\left(n\right)+t_{⊥}\left(n\right)-1$. (**f**). Illustration demonstrating that at transition between two blocks, the persistence time $t_{\|}\left(n\right)$ becomes as long as the trajectory itself. (**g**). Computation of $t_{|}$ (red), $t_{⊥}\left(n\right)$ (purple), and $t_{\|}\left(n\right)$ (cyan) based on (**c**). (**h**). Block invariant ν(n) (blue) computed over time based on (**g**) against the threshold value $\nu_{c}=0.75$; green rectangles underline misclassified trajectory portions. (**i**). Classified trajectory, where black represents free portions and different colours represent distinct detected trapped portions.

We consider either 2 or 3-dimensional trajectories composed of *N* successive coordinates $\left\{x_{1},\ldots ,x_{N}\right\}$, where bold face emphasises the multi-dimensionality of each data point. To make our analysis independent of the trajectory scale, trajectory increments (one-step displacements) are rescaled on each coordinate by their empirical standard deviation. Therefore, the results obtained for Brownian motion are independent of its diffusion coefficient. A recurrence matrix is then calculated from the distance between each pair of points within the trajectory. For each trajectory (see Figure 1a), we construct a positive matrix with Gaussian weights (see Figure 1b):

(1) $$M_{i,j}=\exp\left(-\frac{1}{2}{\left(\frac{|x_{i}-x_{j}|}{\lambda }\right)}^{2}\right),$$

where $|x_{i}-x_{j}|$ denotes distance between two points *i* and *j*, and λ is the test lengthscale. Each element $M_{i,j}$ is in the range [0,1], taking values close to 0 when the distance $|r_{i}-r_{j}|$ is larger than λ. The weights are chosen to be Gaussian in such a way that each element, $M_{i,j}$, remains close to 1 for $|x_{i}-x_{j}|/$λ<1 and decays fast when $|x_{i}-x_{j}|/$λ>1. Therefore, the presence of a trapped portion of a trajectory of size ≈λ translates in the matrix *M* as a square block of near 1 entries whose diagonal is aligned to the matrix diagonal. Due to the random nature of molecule displacement, $M_{i,j}$ entries are noisy, making it difficult to determine the transition between different phases of motion. To overcome this, a local smoothing of the matrix is performed. This operation can be done with computational efficiency by convolving the matrix *M* by a normalized and constant square matrix (2μ+1)×(2μ+1), where μ is the smoothing parameter, through a fast Fourier transform (FFT). An advantage of this is to greatly limit the effect of outliers, such as one-step large jumps in position due to tracking errors within a particle’s trajectory. Whereas locally averaging trajectory coordinates would greatly disturb the shape of the trajectory and enhance the effect of outliers, zeroes in the Laplacian matrix induced by an outlier are removed by local averaging if an outlier lies inside a trapping block.

The smoothed recurrence matrix is then thresholded to obtain a binary matrix *B* by setting to one all the values larger than a critical value $p_{c}$ (see Figure 1c). Here, we choose the critical value to be $p_{c}=\exp\left(-1\right)$, so two points of a trajectory are considered colocalizing if they are within a distance $\lambda \sqrt{2}$ from each other. The consequence of these manipulations turns the problem of finding trapped regions in the trajectories into finding square block structures along the diagonal of the binary matrix *B*.

From matrix *B*, one has to identify the individual block structures. This could be achieved by employing a clustering algorithm, such as k-means or k-medoids algorithms; however, these require known numbers of clusters. Even though empirical methods exist to estimate the number of clusters, such as the ‘elbow’ or ‘silhouette’ methods, they do not perform well when clusters are of a greatly differing number of entities [29]. Although spectral clustering [29] does not suffer from these limitations on cluster sizes, detection of cluster numbers relies on spectral gap detection, which fails when blocks overlap. Thus, it would not be suited for situations where a molecule jumps from one trap to another (Hop-diffusion, described in [10]). We therefore introduce a new methodology that is specific to the detection of block structures and solves all of the aforementioned issues.

We wish to detect if any trajectory step n∈[1,…,N] is a part of a block or not (i.e., trapped or not). For this purpose we define three measures that can be constructed from each point along the matrix’s diagonal $B_{n,n}$ (see Figure 1d for visual illustration). (i) $t_{|}\left(n\right)$: The block time, which is the approximate trapping duration seen from the *n*-th trajectory coordinate. It is computed as the number of matrix elements being both equal to 1 and connected to $B_{n,n}$ along the vertical line $B_{n\pm k,n}$. (ii) $t_{⊥}\left(n\right)$: The neighbouring time, which is related to the size of the window $2t_{⊥}\left(n\right)+1$ centred on time point *n* for which all points colocalize. The neighbouring time is computed as the number of connected matrix elements being equal to 1 along the line perpendicular to the matrix diagonal and going through $B_{n,n}$. (iii) $t_{\|}\left(n\right)$: The persistence time, which is the segment formed by connected matrix elements being equal to 1 that are parallel to the matrix diagonal and starting from the extremity of the segment used to compute $t_{⊥}$. This determines how many *m* frames in the future the lower bound $t_{⊥}\left(n\right)\leq t_{⊥}\left(n+m\right)$ holds.

Let us consider an ideal case where the whole trajectory is trapped such that the recurrence matrix is an N×N square with matrix elements being equal to 1 everywhere. From these three measures, one can deduce an invariant quantity that is valid for any point along the matrix diagonal (see proof in Appendix A):

(2) $$\nu \left(n\right)=\frac{t_{|}\left(n\right)}{t_{\|}\left(n\right)+t_{⊥}\left(n\right)-1}=1.$$

Figure 1e illustrates the computed block time as a function of time (red) and how neighbouring (cyan) and persistence (purple) time compensate each other to verify the equality. This equality is a necessary condition for the point $x_{n}$ to belong to a square block. Specific events or features related to trapping interruption will cause violation of this equality.

For an ideal free portion, the matrix is diagonal (let us call it 1−diagonal). In the case of a trapping event followed by free diffusion at n+1, there is a sharp transition from ν(n−1)=1 to ν(n+1)=1/N. Let us consider a special case, where two successive trapping events are spatially separated such that their corresponding blocks, of size $s_{1}$ and $s_{2}$, respectively, share only a single point (the transition point *n*) that lies on the matrix diagonal. Given that the trajectory is longer than the two trapping events, $N>s_{1}+s_{2}$, there is a sharp transition at *n* because the increase of $t_{\|}$ from $s_{1}-2$ to *N* is not compensated by the increase of $t_{|}$ from $s_{1}$ to $s_{1}+s_{2}-1$ (see Figure 1f). In the case where two trapping events occur successively at even closer locations, their corresponding blocks will overlap. Even though the equality would be broken at the transition point, departure from ν=1 may not be very sharp because the transition point is no longer on the matrix diagonal, and accordingly, the persistence time is bounded by blocks sizes $t_{\|}<s_{1}+s_{2}$. Adding $n_{d}$ diagonal lines along each side of the matrix diagonal, such that an ideal free motion for which *B* is a 1−diagonal matrix would become a $(2n_{d}+1)-$diagonal matrix, helps to enhance the variation in ν at the transition point by changing the bound to $t_{\|}<N-2n_{d}$, where $n_{d}$ is the number of diagonals. Adding sufficient numbers of lines makes the persistence time $t_{\|}\left(n\right)$ almost as long as the trajectory duration itself, so that when the invariant is violated, ν becomes very close to 0.

The number of diagonal lines that should be added depends on the lengthscale λ and the smoothing parameter μ. In general, adding more diagonal lines allows one to distinguish between traps that are very close to each other. In turn, a large number of diagonal lines reduces the precision of change-point detection for isolated traps. In order to decide the number of diagonal lines used, we performed simulations. Block time has been calculated from $M=10^{3}$ simulated trajectories of $N=2\times 10^{3}$ steps drawn from two reference types of motion that mimic free diffusion.

In the first case, we simulated Brownian motion (Bm) as the classical model of a freely moving molecule in a homogeneous medium. In the second case, we simulated subdiffusive, fractional Brownian motion (fBm) [30] with anomalous exponent α=0.7 (Hölder exponent H=0.35) as a prototypical diffusion in a crowded environment at percolation threshold [31,32,33,34]. In both cases, trajectories were simulated in both 2 and 3 dimensions. As a compromise between sensitivity and precision, the tenth percentile values of block time are used for the rest of the paper for the numbers of diagonal lines to be filled, independent of the dimension of the problem and of the user’s choice of reference model (see Appendix D for a comparison of the effect on detection results of the number of added lines).

It is possible that our invariant is broken because of lacunarities inside blocks due to the random nature of molecules’ displacements, which can easily be avoided by filling lacunarities inside block components along the diagonal (function imfill in MATLAB). Figure 1g presents the three measures for the trajectory in Figure 1a. The graph shows that inside a block, the pattern is very similar to the one presented in Figure 1e for the ideal case and shows the large change in persistence time at a block transition.

We claim that the *n*-th point of the trajectory is in a block when ν(n) is larger than a critical value $\nu_{c}$. In practice, blocks are never perfect squares, so we choose $\nu_{c}=3/4$ as a criterion such that blocks can be deformed as illustrated in Figure 1h. However, even in the case of purely free motion (e.g., Brownian motion), some blocks would still be detected because it takes a random finite time to escape a region of size λ. To ensure that a detected block is due to trapping and not due to chance, we chose a *p*-value approach. For each test lengthscale and each type of test motion (2D and 3D Brownian motion and fractional Brownian motion), we simulated $10^{3}$ trajectories and computed the matrices $B_{ij}$ before adding diagonal lines based on our previous simulations. Those simulated trajectories were very long ($10^{4}$ steps each) in order to ensure the capture of very large potential blocks as test lengthscale λ increases. Block size was computed as the number of consecutive points for which our criterion $\nu \left(n\right)>\nu_{c}$ is verified. From these simulations, we estimated in each case the empirical cumulative probability density of block size. From a given *p*-value $p_{val}$, the hypothesis that a detected block is a real trapping event (compared to the reference simulated motion: Bm or fBm) is then rejected if the cumulative probability density associated with the tested block size is smaller than $1-p_{val}$.

## 3. Simulations

In this section, we present performance tests for our algorithm in 2D and 3D and compare it to an alternative algorithm, DC-MSS [16], where possible (in 2D).

### 3.1. Fixed Parameters

For the analysis, the smoothing parameter μ, the number of lines to be filled along the matrix diagonal, and the *p*-value needed to be set. We chose μ=2 in such a way that high-frequency variability in the matrix $M_{ij}$ would be dampened without significantly affecting the precision of change-point detection. Then, based on simulations on the effect of different additional diagonal lines (see Appendix D), we added a number of diagonal lines corresponding to the tenth percentile of block-times. Finally, reasoning that the tail of a Brownian motion’s first-passage-time distribution from the centre to the border of a disk spans over multiple timescales, choosing a *p*-value very close to 1 would exclude many transient trapping events. Accordingly, we fixed the *p*-value $p_{val}=0.05$ as a compromise between the sensitivity and reliability of the method and then varied simulation parameters to assess the potential of our approach.

### 3.2. Simulation

To test our methodology, for each data point presented below, we simulated $10^{3}$ of either 2D or 3D trajectories of $10^{3}$ steps each. Molecules alternate between a free diffusive state and a trapped state in which the molecule remains within a region of set size. We chose the free state to be Brownian motion with one-step diffusion lengthscale σ=1, corresponding to a diffusion coefficient D=1/2. The trapped state was chosen to be reflected Brownian motion inside a disk (2D) or sphere (3D) of radius *R* with the same diffusion coefficient. Similarly, we produced another dataset (2D and 3D), where instead of Brownian motion, we modelled free portions with fractional Brownian motion with Hölder exponent H=0.35, corresponding to an anomalous exponent α=0.7. In all cases, the random duration of each state was chosen to be Poisson distributed with mean $\tau_{Bm}$ and $\tau_{trap}$ for the free and trapped states, respectively. White noise with standard deviation $\sigma_{err}$ was added to trajectory coordinates to model the effect of the localisation error, starting from low noise $\sigma_{err}=\sigma /10$ to mild noise $\sigma_{err}=\sigma /2$ and finally strong noise with an equivalent standard deviation of trajectory one-step displacements $\sigma_{err}=\sigma$.

### 3.3. Results

**Figure 2:**
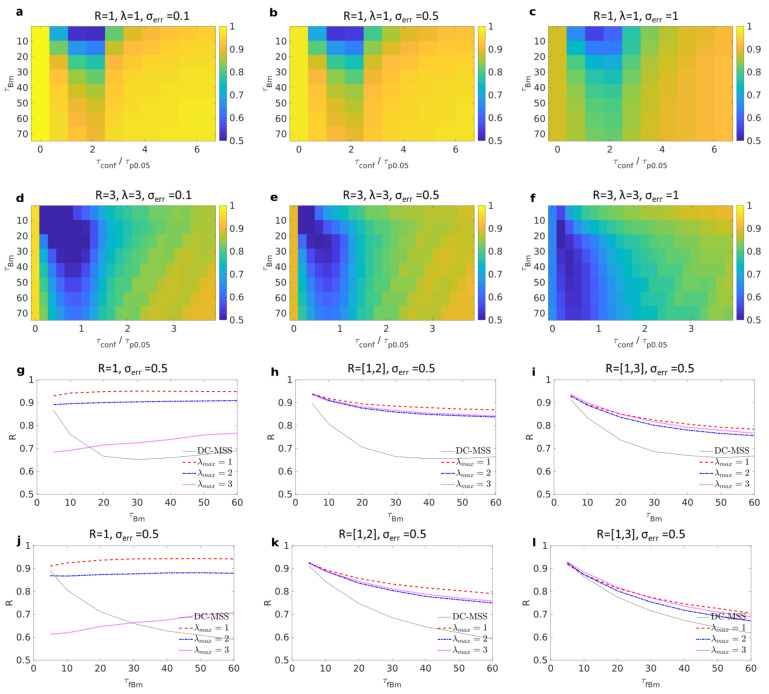
Each panel presents the recognition score ∈[0,1] for 2D trajectories alternating between free and trapped motions. (**a**–**c**) Trapping radius is R=1, and test lengthscale is λ=1. Shown are tested combinations of free Brownian motion of mean duration $\tau_{Bm}=\left[5,\;10,\;20,\ldots ,\;70\right]$ and mean trapping duration $\tau_{conf}\in \left[0,\;60\right]$; coordinates are perturbed with white noise of level $\sigma_{err}=\sigma \times \left[0.1,\;0.5,\;1\right]$ (**a**–**c**). (**d**–**f**) R=3 and λ=3. Each rectangle represents a combination of free Brownian motion of mean duration $\tau_{Bm}=\left[5,\;10,\;20,\ldots ,\;70\right]$ and mean trapping duration $\tau_{conf}\in \left[0,\;210\right]$; coordinates are perturbed with white noise of level $\sigma_{err}=\sigma \times \left[0.1,\;0.5,\;1\right]$. (**g**–**i**) Free motion is Brownian motion; noise level $\sigma_{err}=0.5\sigma$ was added to trajectories. Trapping radius is in the range $R\in [1,R_{max}]$, where $R_{max}=1,2,3$ in (**g**–**i**). In each case, test lengthscales from 1/2 to $\lambda_{max}$ by increments of 1/2 are combined where $\lambda_{max}=1,2,3$ (dashed red, dotted-dashed blue, and dotted magenta). Black line shows the recognition score obtained from DC-MSS algorithm. (**j**–**l**) Same as for (**g**–**i**) except that the free motion is replaced by subdiffusive fractional Brownian motion with Hölder exponent H=0.35.

Figure 2a shows the results where both the time spent in free duration and in trapping were varied while the trapping radius was always R=1, and the test lengthscale was λ=1. Different levels of noise $\sigma_{err}=\sigma /10,\;\sigma /2,\;\sigma$ were added, respectively, in Figure 2a–c. In these cases, the minimal duration for detecting a trapping event is $\tau_{p0.05}=9$ frames (see table in Appendix E). In these three cases, when there is no confinement at all ($\tau_{conf}/\tau_{p0.05}=0$), the recognition score is close to 1, meaning that the algorithm is robust to false negatives and is able to confirm the absence of trapping. In most cases, more than 90% of trajectories are correctly assigned to their state. The method performs poorly when the trapping duration is close to or shorter than $\tau_{p0.05}$ or when the time spent between two trapping events is smaller than the time it takes to explore a distance larger than the trap size. Both mild and strong noise does lower the recognition score, but only marginally. Figure 2d–f test cases when radius R=3 and the test lengthscale is λ=3. In this case, the conclusions are the same, but one has to keep in mind that the durations are much longer because the minimum duration for detecting trapping with λ=3 is $\tau_{p0.05}=42$ (see table in Appendix E).

The above presented cases are idealised because, except when searching for a particular trap size, one does not precisely know the size of traps a priori. A reasonable range can instead be determined by observation of the experimental data. Taking advantage of the robustness to false negatives offered by our *p*-value approach, we propose combining the recognition for each lengthscale into a single one. We combine results by taking the union of detected trapped frames, considering lengthscales in the range $\lambda \in [1,\lambda_{max}]$ by increments of 0.5. We simulated trajectories alternating between free motion and trapping of distributed sizes. Possible trap radii are uniformly distributed in the range $\left[1,R_{max}\right]$, where $R_{max}=1,2,3$ in Figure 2g–i, respectively. The duration in each trapped state is set to be $\tau_{conf}=6R^{2}+50$, so the trapping time takes into account the radius of the trapping area plus an offset of 50 frames. Trapping was simulated as reflected Brownian motion with an integration step dt=1/2 unless the diffusion length during a step was larger than a third of the radius $\sqrt{2Ddt}>R/3$, in which case positions were approximated as being uniformly distributed inside the trap.

For each of these three cases, noise level $\sigma_{err}=0.5\sigma$ was added to trajectories, and we then tested our combination scheme with three possible $\lambda_{max}=1,2,3$. For comparison, we applied the DC-MSS algorithm [16] to our simulated data with the default parameters. DC-MSS separates the data into four categories: immobile, confined, free, and superdiffusive. To make it comparable to our scheme, we considered the two first categories as being ‘trapped’ and the two latter as being ‘free’. In Figure 2g the performance of DC-MSS is better than ours when we overestimate the maximum test lengthscale $\lambda_{max}=3$, which overestimates three times the maximum trap size $R_{max}=1$. In turn, choosing $\lambda_{max}=2$ already significantly improves our classification, and $\lambda_{max}=1$ gives close to perfect recognition. Then, in cases $R_{max}=2$ (Figure 2h) and $R_{max}=3$ (Figure 2i), DC-MSS had a consistently lower score for any choice of parameters $\lambda_{max}$. It can be surprising that in Figure 2h,i, $\lambda_{max}=1$ outperforms the other $\lambda_{max}$ in all cases, while the size of the traps can be larger than this. We explain this by the fact that the test lengthscale does not specify the trap size to be discovered and instead describes distances between points to be considered ‘in the vicinity’. When a molecule spends enough time inside a trap of radius R=3, then even with $\lambda_{max}=1$, any trajectory point will colocalize with many other points in such a way that the recurrence matrix $M_{i,j}$ will be in ‘quasi-block’ form (a block with many holes). In this case, the combination of the smoothing step and the lacunarities-filling will complete the block and allow for accurate detection. In turn, larger lengthscales $\lambda_{max}$ will tend to include, along with a trap, some free points in the vicinity of the confinement area, thus lowering the recognition score.

**Figure A1:**
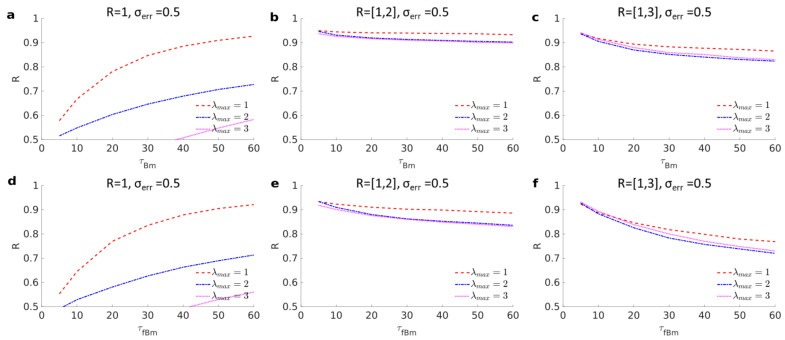
Each panel presents the recognition score ∈[0,1] for 3D trajectories alternating between free and trapped motions. (**a**–**c**) Free motion is Brownian motion with added noise level $\sigma_{err}=0.5\sigma$. Trapping radius is in the range $R\in [1,R_{max}]$, where $R_{max}=1,2,3$ in (**a**–**c**). In each case, test lengthscales from 1/2 to $\lambda_{max}$ by increments of 1/2 are combined, where $\lambda_{max}=1,2,3$ (dashed red, dotted-dashed blue, and dotted magenta). (**d**–**f**) Same as for (**a**–**c**) except that the free motion is replaced by subdiffusive fractional Brownian motion with Hölder exponent H=0.35.

We also considered the case in which trajectories alternate between subdiffusive fractional Brownian motion and trapping. In the case of a single trap size, results were similar to those obtained in Figure 2a–f (data not shown). In the case of multiple traps’ radii (see Figure 2j–l) similar results were obtained, meaning that our approach can distinguish subdiffusion due to molecular crowding from actual trapping in a nano-domain. In comparison, the DC-MSS algorithm tends to misclassify free portions as being trapped, thus giving lesser scores. In Appendix C, additional simulations performed in 3D gave similar results for both diffusive Brownian motion and subdiffusive fractional Brownian motions as ‘free states’ (see Figure A1).

Lastly, we verified that trajectory duration has only negligible effects as long as trajectory duration is longer than the minimum duration for trapping detection (not shown).

## 4. Application to Experimental Data

**Figure 3:**
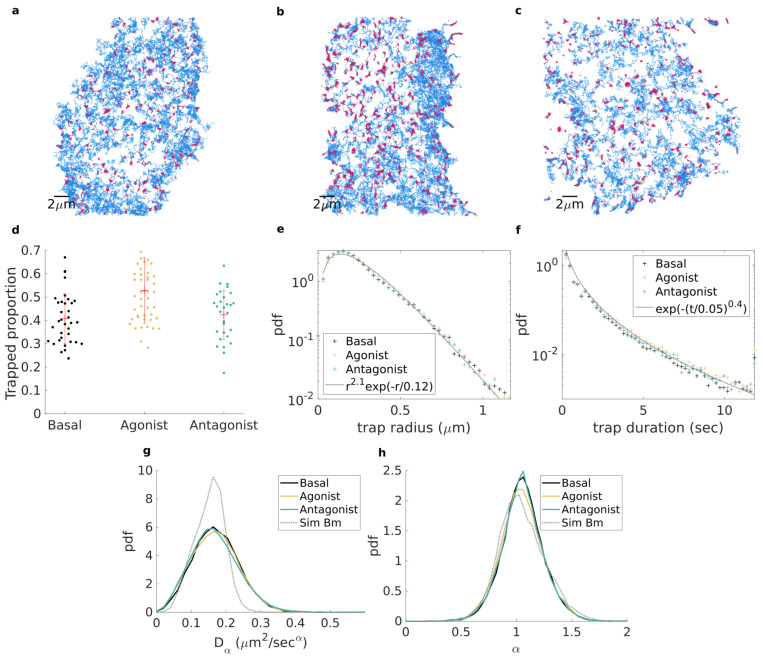
(**a**–**c**). All receptor trajectories longer than 50 frames from a single cell in each group; trajectory portions are coloured according to whether they are detected as free (blue) or trapped (red). Cells are, respectively: (**a**) in basal state, (**b**) stimulated with agonist, and (**c**) treatment with neutral antagonist. (**d**). Proportion of trapped molecules per frame; each point corresponds to a cell for basal (black), agonist stimulated (yellow), and neutral antagonist treated (green). (**e**,**f**). Empirical probability density function for basal (black), agonist stimulated (yellow), and neutral antagonist treated (green) of (**e**) trap radius and (**f**) trapping duration. Grey lines denote fitting with Gamma distribution (**e**) and stretched exponential (**f**). (**g**,**h**). Empirical probability density estimated for free trajectory portions longer than 50 frames of (**g**) the anomalous diffusion exponent α and (**h**) the corresponding generalized diffusion coefficient $D_{\alpha }$.

Based on our methodology, with λ=[0.5,1,1.5,2], smoothing parameter μ=2 and $p_{val}=0.05$, and subdiffusive fBm as our reference for free motion, we investigated the effect of different drugs on the diffusion and trapping of $\beta_{2}$ adrenergic receptor ($\beta_{2}$AR) on the plasma membrane. We recorded fluorescently labelled $\beta_{2}$AR molecules with total internal reflection microscopy, as they diffuse in the plasma membrane of living cells (2D recording) (see Appendix B for experimental methods). We first characterized receptors under basal conditions (36 cells), without pharmacological stimulus. Next, we treated the cells with a gold-standard agonist (isoproterenol) that activates receptors (47 cells). Additionally, we probed receptors with a neutral antagonist (propranolol), which prevents ligand-dependent receptor activation (29 cells). Figure 3a–c show, respectively, all of the trajectories longer than 50 frames (for improved visibility) from a single cell for each described treatment. Portions of trajectories are coloured according to their identified state (trapped in red and free in blue).

It clearly appears that, although trapping is present in all cases, the prevalence of trapping is increased upon agonist stimulation. This is quantitatively supported in Figure 3d, where it is shown that under basal conditions, 39.2% of receptors at each frame were trapped on the plasma membrane. Upon agonist stimulation, this percentage increased to 52.2%, while it remained similar (45.5%) after neutral antagonist treatment. To test the relevance of the observed change, we used a non-parametric Kruskal–Wallis test with Tukey–Kramer correction for multiple comparisons. We found the change between basal and agonist stimulation to be significant ($p=2\times 10^{-4}$), clearly demonstrating an effect of agonist stimulation on receptor diffusion dynamics. Contrarily, the change between basal and neutral antagonist treatment was not significant (p=0.74, while the difference between agonist and neutral antagonist was significant ($p=9\times 10^{-3}$), suggesting that the drug employed directly influences the receptor trapping, an increase in which correlates with activation of the receptors.

We then sought to further explore the differences observed between these cases. For each trapped trajectory portion, we computed the trapped radius as the distance from the estimated centre of the trap (evaluated as the median of *x* and *y* coordinates for a trapped portion) and the point further away than 95% of points within the trapped portion. In Figure 3e, we binned all of the trapped radii into an empirical probability density function (pdf) which was revealed to be similar for the three conditions, suggesting that the trapping domains are of the same nature in all cases. In all cases, the pdf of trapped radii could be fitted approximately with a Gamma distribution, highlighting the exponential decay of the tail of the distribution. This was further reinforced by the computation of the empirical pdf of trapped portions’ durations (see Figure 3f), from which we again obtained a similar empirical pdf for all three conditions. The tails of the trapping duration pdf were fitted to a stretched exponential distribution, thus encompassing the wide (yet finite) range of trapping durations.

Finally, we enquired into the dynamics of free trajectory portions. To do so, following [35], we computed the time-averaged mean square displacement (TAMSD) of each portion on each coordinate as

(3) $$\delta^{2}\left(n,N\right)=\frac{1}{N-n}\sum_{k=1}^{N-n}{\left(x_{k+n}-x_{k}\right)}^{2},$$

and summed the result for both coordinates before performing a non-linear fitting, over the lag-time range n∈[1,5], with the formula for ensemble-averaged TAMSD for a 2D ergodic anomalous diffusion process (e.g., fractional Brownian motion), with localisation error $\sigma_{err}$

(4) $$\langle \delta^{2}\left(n,N\right)\rangle =4D_{\alpha }n^{\alpha }+4\sigma_{err}^{2},$$

where α is the anomalous exponent, and $D_{\alpha }$ is the generalized diffusion coefficient. From this, we obtained the empirical pdf for both anomalous exponent and generalized diffusion coefficients for each condition and observed once again that it was remarkably consistent among the tested conditions. The exponents for free portions of trajectories (see Figure 3g) were distributed slightly over α=1 (average exponent 〈α〉= 1.04, 1.05, 1.04), corresponding to simple Brownian motion. The generalized diffusion coefficients were very similar in all tested conditions (see Figure 3h) with an average $\langle D_{\alpha }\rangle =$ 0.173, 0.169, 0.168 μm${}^{2}$ s${}^{-1}$ for basal, agonist, and antagonist, respectively. For comparison, we computed the pdf of exponent and $D_{\alpha }$ from simulated Brownian motion (see Figure 3g,h), using the same parameters for trajectory duration and mean diffusion coefficient as the free portions found in the case of the tested agonist. The distributions obtained from simulations match the experimental for exponent (average exponent from simulation is $\langle \alpha_{sim}\rangle =1.05$). However the experimental distributions of diffusion coefficients are wider that the simulated one. We conclude that the distributed nature of the estimated exponent is mainly due to the intrinsic randomness of the TAMSD applied to random trajectories [36,37] while the spread of $D_{\alpha }$ highlights the heterogeneous nature of cell membrane.

Altogether, these results shed light on the effects of different drug treatments on receptor dynamics. We observe that receptors do not slow down after agonist stimulation. In fact, the change we observe is that receptors are more likely to be trapped, with the nature of the trapping domains remaining the same. For the case of the antagonist, we do not find a significant difference compared to the basal condition, which correlates with the proposed model where neutral antagonists impart no intrinsic activity on the receptor in the absence of an accompanying agonist. We conclude that on timescales longer than our exposure time frame (30 ms), receptors alternate between free lateral diffusion that could be modelled by Brownian motion with fluctuating diffusion coefficient [38,39,40,41,42,43,44,45,46,47,48,49] and transient trapping in nano-domains of distributed size.

## 5. Conclusions

In conclusion, we present an algorithm (Code availability: MATLAB code can be downloaded from https://github.com/YannLanoiselee/Transient_trapping_analysis, accessed on 9 August 2021) that can accurately detect transient trapping events from a single trajectory either in two or three dimensions. Our approach is based on recognizing block structures along the diagonal of a thresholded, smoothed recurrence matrix. To this end, we introduced three local measures to be computed along the diagonal of the matrix from which we deduced an invariant quantity inside blocks (trapped portions).

Then, based on a set of user-inputted test lengthscales and on simulations of Brownian and fractional Brownian motions in 2D and 3D as reference processes, we could assess the minimal size of blocks that could be interpreted as the molecule actually being trapped and not a block due to chance, depending on a *p*-value. We tested our method on a set of simulated data and verified the good performance in 2D and 3D when the free type of motion is either Brownian motion of sub-diffusive fractional or Brownian motion with anomalous exponent α=0.7. We checked the robustness of our results against increasing magnitudes of localisation error. We also compared our 2D results with the classification obtained from the DC-MSS algorithm [16] and showed that our method is more accurate in the task of detecting trapping in all tested cases.

Finally, we applied our analysis to single-particle trajectories of $\beta_{2}$ Adrenergic G-protein-coupled receptors recorded through total internal reflection microscopy. Three conditions were tested: the basal state, stimulated with an agonist, and treatment with a neutral antagonist. In all cases, we found that molecules explore traps with similar distributions of size and duration. Instead, it was only the frequency with which molecules were trapped that was different. TAMSD analysis of the free portions of trajectories led to the conclusion that molecules were mostly undergoing Brownian motion, with a variety of parameters indicative of cell membrane heterogeneity. The demonstration of this technique on real biological data and delineation of pharmacological principles using it (agonist = activation, antagonist = net 0 effect) suggest that our methodology to detect trapping events can be used to study the complexity of both intracellular (3D) and membrane proteins (2D) in live cells.

## Figures and Tables

**Figure A2:**
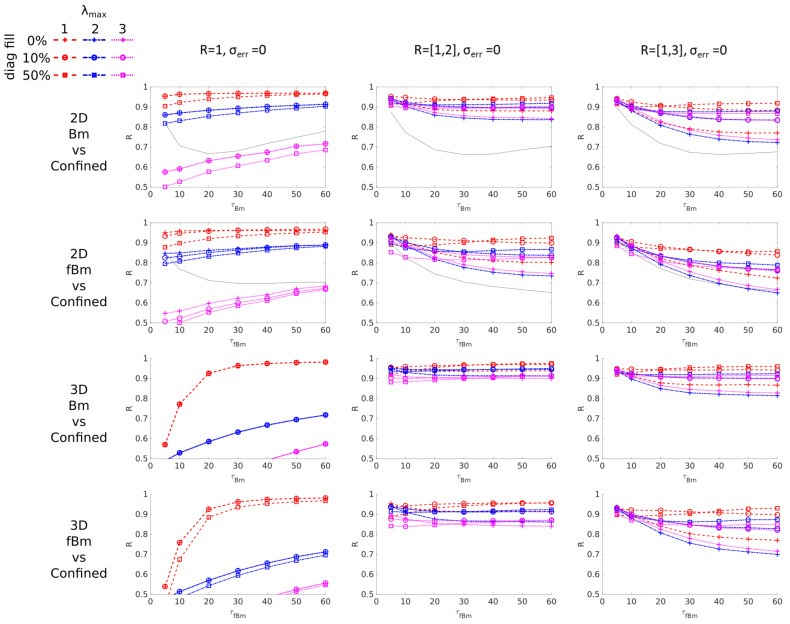
Each panel presents the recognition score ∈[0,1] for 2−3D trajectories alternating between free and trapped motions. Each column corresponds to a trapping radius range $R\in [1,R_{max}]$, where $R_{max}=1,2,3$, respectively. Rows corresponds to different dimensionality and types of free motion 2D and Bm, 2D and fBm, 3D and Bm, and 3D and fBm, respectively. For the two first rows, black lines correspond to predictions from DC-MSS. Red, blue, and magenta lines correspond to $\lambda_{max}=1,2,3$, while crosses, circles, and squares indicate that lines along the diagonal of the binary matrix *B* have been filled according to the zeroth, tenth, and median percentiles of block times computed from simulations for either Bm or fBm according to the situation.

## Data Availability

The data presented in this study are openly available in Lanoiselée, Yann; Grimes, Jak; Koszegi, Zsombor; Calebiro, Davide (2021): Trajectory of individual of beta-2 adrenergic receptors at the plasma membrane of Chinese hamster ovary cells (CHO-K1) obtained from TIRF microscope. figshare. Dataset. (https://doi.org/10.6084/m9.figshare.15157410, accessed on 9 August 2021).

## Appendix A. Proof of the Square Block Invariant

To prove the equality in Equation (2), we proceed by two inductions. For a square block of fixed side length *c*, we start by noting the symmetry with respect to the line perpendicular to the matrix diagonal going through point c/2 (that lies between two points for odd *c*). Then, we define n∈[1,c/2]; our relationship is verified for n=1, and we suppose it true for *n*. Then, observing that $t_{|}\left(n+1\right)=t_{|}\left(n\right)$, $t_{\|}\left(n+1\right)=t_{\|}\left(n\right)-2$, and $t_{⊥}\left(n+1\right)=t_{⊥}\left(n\right)+2$, we deduce ν(n+1)=ν(n)=1. For the second induction, we start by noting that our relationship is valid for c=1, and we suppose it is true for c=k. Then, for c=k+1, we find that $t_{⊥}\left(n\right)$ remains unchanged, while both $t_{|}\left(n\right)$ and $t_{\|}\left(n\right)$ increase by one, thus again verifying our equality. The relationship is thus valid for arbitrary block sizes and at any point along the diagonal within the block.

## Appendix B. Experimental Methods

### Appendix B.1. Materials

Cell culture reagents, Lipofectamine 2000, and TetraSpeck fluorescent beads were purchased from Thermo Fisher Scientific. Isoproterenol and Propranolol were from Tocris Bioscience. The fluorescent SNAP-Surface 549 was from New England Biolabs. Ultraclean glass coverslips were obtained as previously described [50]. For single-molecule experiments, Chinese hamster ovary K1 (CHO-K1) cells (ATCC) were cultured in phenol red-free Dulbecco’s modified Eagle’s medium (DMEM)/F12, supplemented with 10% FBS, penicillin, and streptomycin at 37 ${}^{{}^{\circ}}$C, 5% CO${}_{2}$. Cells were seeded onto ultraclean 25 mm round glass coverslips at a density of 3 × 10${}^{5}$ cells per well. On the following day, cells were transfected using Lipofectamine 2000 with N-terminally SNAP-tagged human $\beta_{2}$AR (SNAP-$\beta_{2}$AR) [50] and N-terminally GFP-tagged clathrin light chain (GFP-CCP) (kindly provided by Emanuele Cocucci and Tom Kirchhausen), following the manufacturer’s protocol. Cells were labelled with 1 μM SNAP-Surface 549 in complete culture medium for 20 min at 37 ${}^{{}^{\circ}}$C and imaged by single-molecule microscopy ≈4 h after transfection to obtain low physiological protein expression levels [2,50]. Cells were washed with complete culture medium and imaged in Hank’s balanced salt solution (HBSS) supplemented with 10 mM HEPES. The labelling efficiency was ≈90% ([50]) with non-specific labelling <1%. $\beta_{2}$ARs were stimulated with either 10 μM Isoproterenol or treated with 10 μM Propranolol.

### Appendix B.2. Single-Molecule Microscopy

Single-molecule microscopy experiments were performed using total internal reflection fluorescence (TIRF) microscopy on a custom system, based on an Eclipse Ti2 microscope (Nikon, Japan) equipped with a 100× oil-immersion objective (NA 1.49, Nikon); 405, 488, 561, and 637 nm diode lasers; an iLas TIRF illuminator; quadruple band excitation and dichroic filters; a quadruple beam splitter; 1.5× tube lens (Cairn Research); four EMCCD cameras (iXon Ultra 897, Andor); and hardware focus stabilization. The sample and objective were maintained at 37 ${}^{{}^{\circ}}$C throughout the experiments. Multicolour single-molecule image sequences were acquired simultaneously at full frame in frame transfer mode, corresponding to one image every 30 ms. Automated single-particle detection and tracking were performed with the u-track software [51], and the obtained trajectories were further analysed using custom algorithms in MATLAB environment as previously described [2].

## Appendix C. Simulations for the 3D Case

In this section, we present the simulation results obtained in three dimensions.

## Appendix D. Effect of the Number of Diagonal Filled

In Figure A2, we analysed 2D and 3D simulated trajectories alternating between either Bm or fBm and reflected Brownian motion. Then, we computed the recognition score for three possible maximum lengthscales $\lambda_{max}=1,2,3$. Then, for each of these $\lambda_{max}$, we computed results depending on the number of lines that were added along to the matrix diagonal of the binary matrix *B*. We considered no diagonal added at all ($d_{0}$) or the tenth percentile $d_{10}$ or median $d_{50}$ of the block time obtained from simulations of Bm or fBm in either 2D or 3D. In all considered cases, the best maximum lengthscale was $\lambda_{max}=1$, and the best recognition score was obtained with $d_{10}$.The case $d_{0}$ was similar to $d_{10}$ for $R_{m}ax=1$ but failed for larger trapping radii. On the other hand, $d_{50}$ could not capture change-points for a short duration of the free states as well as $d_{10}$.

## Appendix E. *p* Value Tables

In this section, we present the minimal trapped duration including the number of filled diagonal lines (for $d_{10}$) corresponding to *p*-values [0.1,0.05,0.01] for different test lengthscales λ for fixed smoothing parameter μ=2 and $\nu_{c}=0.75$ (see Table A1). Values corresponding to each test lengthscale λ are obtained from $10^{3}$ simulated trajectories of $10^{4}$ steps. Simulations have been performed on 2-dimensional Brownian motion with diffusion coefficient D=1/2 (although the result is independent of *D* because of rescaling) and on 2-dimensional fractional Brownian motion (each coordinate generated independently) with diffusion coefficient D=1/2 and a Hölder exponent H=0.35 corresponding to an anomalous diffusion exponent α=0.7, similar to what is found in diffusion in a crowded molecular environment.

In the case of 3D diffusion (see Table A2), similar simulations have been performed with one extra dimension. A table of the minimal trapped duration including the number of filled diagonal lines corresponding to *p*-values [0.1,0.05,0.01] can be seen below. They are generally shorter because a trajectory has one more degree of freedom to escape. For Brownian motion, the mean square-displacement is increased 50%.

**Table A1:** Minimal size for a block to be considered a trapped portion for when reference motion is 2D Brownian motion (left) and 2D fractional Brownian motion with H=0.35 (right).

	2D Bm			2D fBm H=0.35		
λ	$p_{val}=0.1$	$p_{val}=0.05$	$p_{val}=0.01$	$p_{val}=0.1$	$p_{val}=0.05$	$p_{val}=0.01$
0.5	4	5	6	5	5	7
1	8	10	14	10	13	18
1.5	17	20	28	28	35	52
2	26	32	46	54	68	103
2.5	38	46	68	88	112	176
3	45	57	87	131	169	272

**Table A2:** Minimal size for a block to be considered a trapped portion for when refence motion is 3D Brownian motion (left) and 3D fractional Brownian motion with H=0.35 (right).

	3D Bm			3D fBm H=0.35		
λ	$p_{val}=0.1$	$p_{val}=0.05$	$p_{val}=0.01$	$p_{val}=0.1$	$p_{val}=0.05$	$p_{val}=0.01$
0.5	4	4	5	4	4	5
1	5	6	8	6	7	8
1.5	10	11	15	13	15	21
2	14	17	23	24	28	40
2.5	21	25	34	39	48	69
3	28	33	46	60	74	109
